# Tracheal extubation under Narcotrend EEG monitoring at different depths of anesthesia after tonsillectomy in children: a prospective randomized controlled study

**DOI:** 10.3389/fped.2024.1344710

**Published:** 2024-03-28

**Authors:** Hongqiang An, Xifeng Zhang, Lingling Chen

**Affiliations:** Department of Anesthesiology, Children’s Hospital of Nanjing Medical University, Nanjing, Jiangsu, China

**Keywords:** depth of anesthesia, tracheal extubation, tonsillotomy, children, cognitive function

## Abstract

**Objective:**

This study aims to investigate whether tracheal extubation at different depths of anesthesia using Narcotrend EEG (NT value) can influence the recovery quality from anesthesia and cognitive function of children who underwent tonsillotomy.

**Methods:**

The study enrolled 152 children who underwent tonsillotomy and were anesthetized with endotracheal intubation in our hospital from September 2019 to March 2022. These patients were divided into Group A (conscious group, NT range of 95–100), Group B (light sedation group, NT range of 80–94), and Group C (conventional sedation group, NT range of 65–79). A neonatal pain assessment tool, namely, face, legs, activity, cry, and consolability (FLACC), was used to compare the pain scores of the three groups as the primary end point. The Mini-Mental State Examination (MMSE) and Montreal Cognitive Assessment (MoCA) scales were used to evaluate the cognitive function of children in the three groups before and after surgery as the secondary end points.

**Results:**

Differences were observed in the awakening time and FLACC scores after awakening among the three groups (*P *< 0.05). Among them, Group A exhibited a significantly shorter awakening time and higher FLACC score after awakening than those in Groups B and C (both *P *< 0.05). The total incidence of adverse reactions in Group B was significantly lower than that in Groups A and C (*P *< 0.05). No significant difference was observed in MMSE and MoCA scores before the operation and at 7 days after the operation among the three groups (*P *> 0.05), but a significant difference was found in MMSE and MoCA scores at 1 day and 3 days after the operation among the three groups (*P *< 0.05). In addition, MMSE and MoCA scores of the three groups decreased significantly at 1 day and 3 days after the operation than those at 1 day before the operation (*P *< 0.05).

**Conclusion:**

When the NT value of tonsillectomy is between 80 and 94, tracheal catheter removal can effectively improve the recovery quality and postoperative cognitive dysfunction of children.

## 1 Introduction

Tonsillar hypertrophy is the leading cause of obstructive sleep apnea–hypopnea syndrome (OSAHS) in children, impacting their growth (1–3). Surgical resection has been recognized as the major therapeutic option in clinical practice. For example, tonsillotomy can serve a therapeutic purpose by removing a portion of the tonsils using cold instruments. It can not only alleviate diseases in children but also retain the basic function of tonsils leading to fewer postoperative complications (4). It should be noted that anesthesia and tracheal intubation are required during the operation. Prior studies have documented that the timing of extubation after an operation can impact the quality of the recovery of children after anesthesia and is associated with the occurrence of postoperative adverse complications (5, 6). Therefore, choosing the appropriate depth of anesthesia for tracheal extubation is of great significance for postoperative rehabilitation.

With the wide application of various advanced medical instruments in the clinical field, Narcotrend anesthesia depth monitors can monitor the intraoperative anesthesia depth in real time and effectively predict the loss and recovery of consciousness of the patients, especially for the characteristics of rapid changes in anesthesia depth in children (7). The Narcotrend anesthesia depth monitoring system offers many advantages, such as perfect data classification, low data fluctuation, faster data processing, simple operation, and non-invasiveness (8). It is used to collect and analyze real-time EEG signals at any position of the head of patients through ordinary ECG electrodes and display the depth of consciousness of patients on a color touch screen after automatic analysis and classification. It can provide real-time reflection of the cerebral cortex function of patients contributing to the reduction of anesthesia complications. Accordingly, the present study was conducted to compare 152 children who underwent tonsillotomy to explore the effect of tracheal extubation at different depths of anesthesia using Narcotrend EEG (NT value) on their recovery quality from anesthesia and cognitive function.

## 2 General data and methods

### 2.1 Study subjects

The subjects of the study were children with obstructive sleep apnea–hypopnea syndrome (OSAHS) who underwent tonsillotomy in our hospital from September 2019 to March 2022. This study was registered in the Chinese Clinical Trial Registry (ChiCTR2200032566) and was reviewed and approved by the Medical Ethics Committee of our hospital (2019KY-065). The parents of the enrolled children signed the informed consent. The primary analysis was based on a modified intention-to-treat (mITT) principle. No imputation was performed for missing data.

The inclusion criteria included (1) children aged 4–12 years falling under the American Society of Anesthesiologists (ASA) classes I–II (7); (2) children without upper respiratory tract infection (URTI) in the past 15 days; and (3) children who voluntarily agreed by themselves, along with their families, to participate in the study.

The exclusion criteria included (1) children with a family history of anesthesia-related complications; (2) children with congenital cognitive impairment; (3) children with congenital heart disease; (4) children with coagulation dysfunction; and (5) children with delayed development/microcephaly.

### 2.2 Methods

Non-participants in the study used a computer to generate random numbers, which were used to randomly divide the participants into three groups. These generated random sequence numbers were placed in opaque envelopes that were randomly selected and opened by another anesthesiologist who was not involved in the study to reveal the groupings before the patients were admitted to the operating room. To exclude bias due to different surgical practices and habits, all patients were from the same group of surgeons. The enrolled patients were divided into the conscious group (Group A; NT value, 95–100), light sedation group (Group B; NT value, 80–94), and conventional sedation group (Group C; NT value, 65–79) according to the NT values when tracheal extubation was performed without stimulus response after the operation.

All children were routinely fasted for 8 h on solid and 2 h on clear liquid before the operation. After arriving at the anesthesia preparation room, the children were prepared for intranasal anesthesia in 30–60 min. Peripheral venous access was established after entering the operating room. All children were treated with inhalation anesthesia and intravenous anesthesia. A Philips monitor (IntelliVue Patient Monitor MX500/MX550) was used to monitor the heart rate, mean arterial pressure (MAP), SpO_2_, PETCO_2_, and axillary temperature routinely. The physical signs were detected, and the depth of anesthesia (NT value) was monitored with the Narcotrend EEG monitor (Narcotrend-Compact, Germany).

Anesthesia induction involved the administration of intravenous infusion-induced anesthesia with 0.01 mg/kg penehyclidine hydrochloride, 0.3 mg/kg dexamethasone, 2.5 mg/kg propofol, 4 μg/kg fentanyl, and 0.05 mg/kg cisatracurium, and endotracheal intubation was performed. All children received preoxygenation and airway management by tracheal catheter. For anesthesia maintenance, sevoflurane, fentanyl, and propofol were used to maintain the NT value in the range of 37–46, and the maintenance medication of anesthesia was withdrawn 10 min before the end of the operation (decision made by the surgeon). None of the medications used in this study were found to affect EEG readings in patients. All children underwent tracheal cannulation with pillows placed under their shoulders and their heads tilted backward. Subsequently, the Davis mouth gag was inserted and fixed. Tonsillotomy was performed after the exposure of the mouth and pharynx and complete exposure of the tonsils. After the operation, the secretions and intraoperative rinsing solution in the mouths of children were suctioned out to prevent inhalation. Subsequently, the children with endotracheal tubes were immediately transferred to the post-anesthesia care unit (PACU). The specialist pediatric anesthesiologist in the PACU performed tracheal extubation in these children under sedation when the muscular tone of the child had recovered, the spontaneous respiration was stable, the oral and tracheal secretions were cleaned up, the respiratory exchange capacity was satisfactory, and no decrease was observed within 5 min (tidal volume ≥8 ml/kg, SpO_2_ ≥ 92% when inhaling air, PETCO_2_ ≤ 45 mmHg).

### 2.3 Outcome measures

(1) Two senior anesthesiologists were assigned to record and perform cognitive assessments. The awakening time (also called the eye opening time, which is defined as the cessation of propofol infusion until the patient's eyes open); face, legs, activity, cry, and consolability (FLACC) score; and adverse reactions (8) to extubation were recorded in all children. Primary end points: FLACC score includes expression, body movement, behavior, crying, and comfort. The observed behaviors of children were compared with the scale to get a score for each item, with a range of 0–2 points. The score is added to the total FLACC score, with a range of 0–10 points. A higher value indicates a more severe pain. Adverse reactions to extubation included hypoxemia, emergence agitation (EA), respiratory depression, laryngospasm, and bucking. Hypoxemia is defined as an insufficient level of oxygen in the blood, characterized by a decrease in PaO_2_ and SaO_2_ or SpO_2_ levels, wherein PaO_2_ falls below the lower limit of normal for peers and SaO_2_ or SpO_2_ < 90% at low altitudes (≤2,500 m) and ≤87% at high altitudes (>2,500 m). The agitation score is divided into five levels: 1, sleep; 2, awake and quiet; 3, irritated and crying; 4, non-stop crying; and 5, severe agitation and disorientation.

Meanwhile, MAP and heart rate were recorded in all children at admission (T1), before induction (T2), before intubation (T3), 1 min after intubation (T4), 3 min after intubation (T5), 5 min after intubation (T6), at the beginning of the operation (T7), during tonsillotomy (T8), during adenoidectomy (T9), at the end of the operation (T10), when awakening (T11), and during extubation (T12).

(2) The cognitive function of children was recorded and compared as secondary end points. Through follow-up visits or phone calls, the cognitive function of the children was assessed by the Mini-Mental State Examination (MMSE) and the Montreal Cognitive Assessment (MoCA) scales before surgery and 1 day, 3 days, and 7 days after surgery, respectively (9, 10). The MMSE includes simple questions and problems in several areas: the time and place of the test, repeating lists of words, arithmetic tasks such as the serial sevens, language use and comprehension, and basic motor skills. The MoCA test is characterized by good concurrent validity and can detect cognitive impairment in different neurological disorders, and is used to assess different types of cognitive abilities, including orientation, short-term memory or delayed recall, executive function or visuospatial ability, language abilities, abstraction, animal naming, attention, and clock-drawing test. Higher scores may indicate a higher cognitive level. The baseline cognitive function was evaluated 1 day before the surgery. The postoperative cognitive function assessment was conducted at 1 day, 3 days, and 7 days after the operation.

### 2.4 Statistical analysis

Based on *a priori* power calculations, a cohort sample size of 30 patients would achieve 80% power to detect a correlation of 0.69 (null hypothesis correlation, 0.15) using a two-sided hypothesis with a significance level of 0.0081 (corrected for six comparisons). For non-parametric data, power calculations for Spearman’s rank-order correlation revealed a required sample size of 41 in each group for achieving 80% power to detect a correlation of 0.5 using a two-sided hypothesis testing with a significance level of 0.0081 (family-wise error adjusted for six comparisons). Finally, 211 patients were enrolled for randomization to account for possible dropouts. SPSS22.0 statistical software was used for data processing and statistical analysis. General data, adverse reactions, and other count data were expressed as percentages and tested by *X*^2^. The baseline characteristics and clinical outcomes of the three groups were compared via the independent *t* test, chi-square test, or Fisher’s exact test as appropriate. The recovery time, pain score, MAP, MMSE, MoCA, and other measurement data were expressed by $\bar{x}\pm s$. A one-way analysis of variance was used for the comparison of data between groups, and the Bonferroni test was used for a two-way comparison between groups. Comparisons of measures at different time points were analyzed using repeated measures ANOVA. *P* < 0.05 was considered as statistically significant. Generalized estimating equations were used to test the associations between exposure variables above and all available composite National Institutes of Health Toolbox Cognition scores across all time points (i.e., baseline, postoperative day 1, and postoperative day 2). This generalized estimating equation approach is useful with population-averaged estimates even with the possibility of skewed distributions and misspecification of correlation structures. Power analysis was conducted using Power Analysis and Sample Size Software (2018; NCSS, LLC., USA, https://ncss.com/software/pass).

## 3 Results

### 3.1 Comparison of general data in children

**Figure 1:**
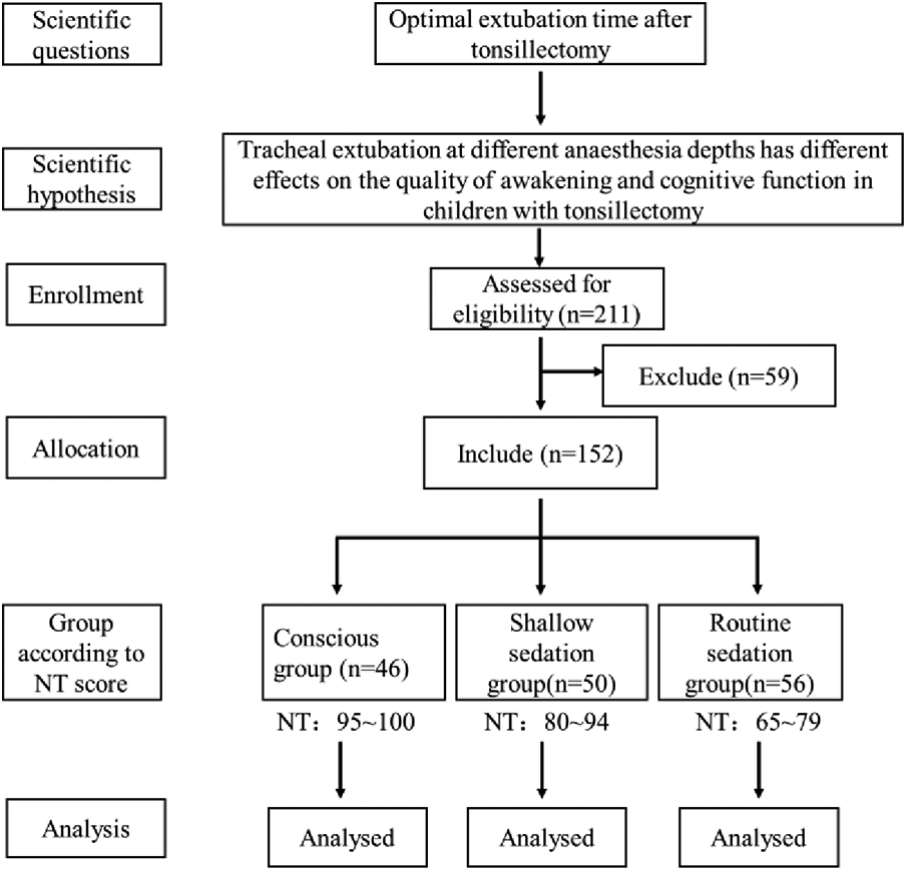
Patient research framework.

From September 2019 to March 2022, 211 children were screened. Among them, 30 were ineligible due to factors such as a family history of anesthesia-related complications (*n* = 7), congenital cognitive impairment (*n* = 12), congenital heart disease (*n* = 10), coagulation dysfunction (*n* = 5), or delayed development/microcephaly (*n* = 11). The final randomization of 152 children underwent tonsillotomy. Fourteen children were excluded from the final data analysis based on the change of surgery procedure and the withdrawal of informed consent. Ultimately, 46 children in Group A, 50 children in Group B, and 56 children in Group C completed the study and follow-up evaluation. The research framework is shown in Figure 1. As shown in Table 1, no significant difference was observed in the comparison of general data such as gender ratio, age, average weight, and operation time (*P *> 0.05) and in all baseline characteristics among the groups.

**Table 1:** Comparison of general data of children ($\bar{x}\pm s$).

Group	*n*	Male	Age (months)	Average weight (kg)	Operation time (min)
Group A	46	24	91.2 ± 24.8	33.2 ± 4.1	22.51 ± 6.1
Group B	50	26	93.4 ± 23.6	32.9 ± 4.6	21.38 ± 7.3
Group C	56	26	92.1 ± 22.5	33.0 ± 4.2	22.86 ± 5.9
*F*		0.213	0.276	0.245	0.226

*F* value: the statistic value of the *F* test.

*P* value: probability, reflecting the possibility of an event.

### 3.2 Comparison of recovery quality of children after extubation

Table 2 presents the differences in the awakening time and pain scores observed after awakening among the three groups (*P *< 0.05). The awakening time was significantly shorter, and the pain score after awakening was significantly higher in Group A than that in Groups B and C (both *P *< 0.05).

**Table 2:** Comparison of awakening time and awakening pain scores of children after extubation ($\bar{x}\pm s$).

Group	*n*	Wake-up time (min)	Pain score
Group A	46	15.4 ± 3.5	6.3 ± 0.8
Group B	50	24.2 ± 4.2*	2.4 ± 0.7*
Group C	56	29.3 ± 4.1^*,#^	2.1 ± 0.5*
*F*		11.231	4.187
*P*		0.001	0.028

* Compared with Group A, *P *< 0.05.

^#^ Compared with Group B, *P *< 0.05.

### 3.3 Adverse reactions after extubation in children

Most children may experience multiple adverse reactions at the same time, such as respiratory depression and hypoxemia. Children with laryngospasm or glossocoma were usually accompanied by hypoxemia, restlessness, or respiratory depression. Table 3 shows that Group A had the highest proportion of restlessness, with an adverse response incidence after extubation of 19 (41.30%). In contrast, the adverse response incidence after extubation in Group B was 5 (10.00%). In addition, Group C had the highest prevalence of hypoxemia, with an adverse response incidence after extubation of 15 (32.61%). Group B experienced considerably fewer adverse effects than those in Groups A and C (*P *< 0.05).

**Table 3:** The person-time of adverse reactions of children after extubation (*n*, %).

Group	*n*	Hypoxemia	Restlessness	Respiratory depression	Laryngospasm	Glossocoma
Group A	46	4 (8.70%)	18 (39.13%)	3 (6.52%)	3 (6.52%)	1 (2.17%)
Group B	50	1* (2.00%)	5* (10.00%)	1 (2.00%)	1 (2.00%)	0 (0%)
Group C	56	10^*,#^ (17.86%)	8* (14.29%)	6^*,#^ (10.71%)	3 (5.36%)	1 (1.79%)

* Compared with Group A, *P *< 0.05.

^#^ Compared with Group B, *P *< 0.05.

### 3.4 Comparison of MAP and heart rate at different time points in children

**Figure 2:**
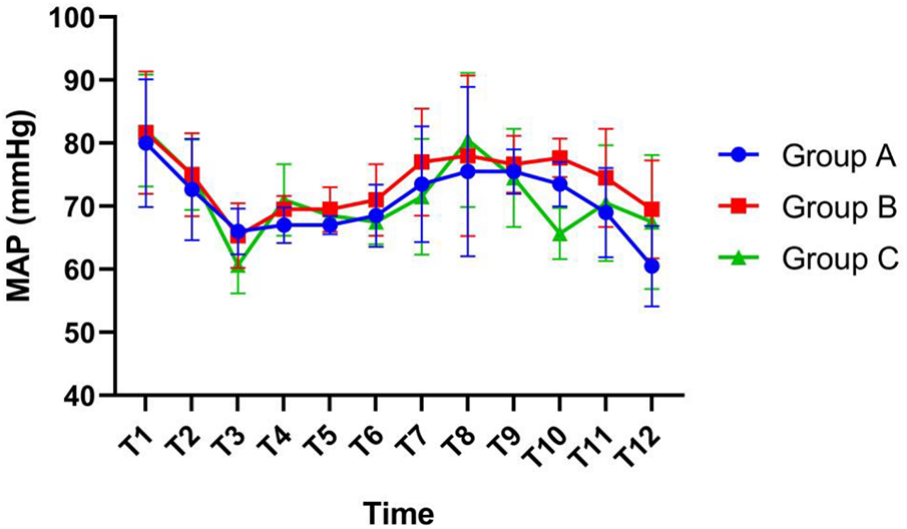
Comparison of mean arterial pressure (MAP) at different time points in children.

No significant difference in MAP was observed at T1–T9 among the three groups. However, at T10, T11, and T12, a difference in MAP among the three groups was noted, with the highest recorded in Group B (*P *< 0.05), as presented in Figure 2.

**Figure 3:**
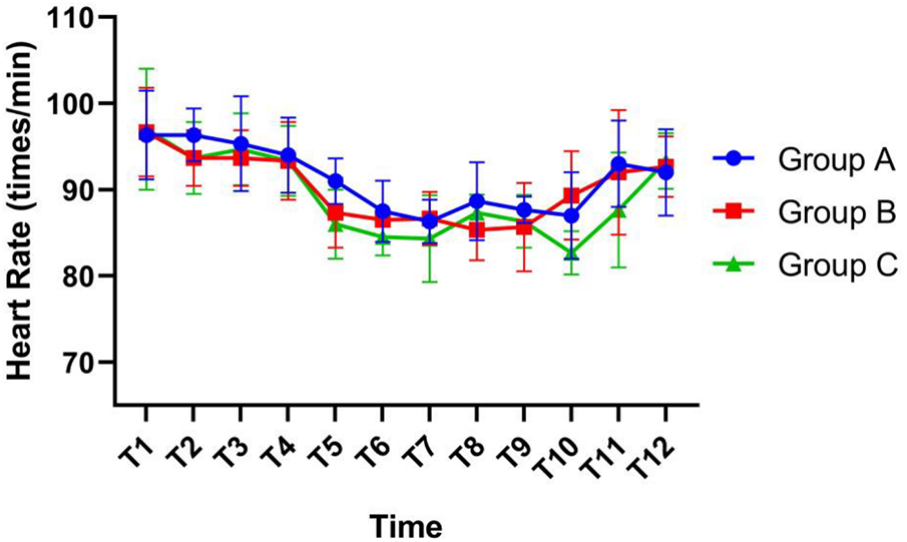
Comparison of heart rates at different time points in children.

At T10 and T11, the heart rates of the three groups were different, and the highest was found in Group B at T10 and Group A at T11. At other time points, there was no significant difference in heart rate, as shown in Figure 3.

### 3.5 Comparison of MMSE and MoCA scores before and after surgery in children

All children were followed up and none were lost to follow-up. No significant difference was observed in MMSE and MoCA scores before the operation and at 7 days after the operation among the three groups (*P *> 0.05). Furthermore, a significant difference was found in MMSE and MoCA scores at 1 day and 3 days after the operation among the three groups (*P *< 0.05). Moreover, MMSE and MoCA scores of the three groups decreased significantly at 1 day and 3 days after the operation than those of the three groups at 1 day before the operation (*P *< 0.05; Tables 4, 5).

**Table 4:** Comparison of Mini-Mental State Examination (MMSE) scores of children before and after surgery.

Group	*n*	1 day before the operation	1 day after the operation	3 days after the operation	7 days after the operation	*F*	*P*
Group A	46	26.67 ± 0.51	21.64 ± 0.40^&^	23.14 ± 0.85^&^	25.84 ± 0.44	7.132	0.003
Group B	50	26.64 ± 0.49	23.24 ± 0.85^*,&^	25.65 ± 0.48^*,&^	25.83 ± 0.48	6.121	0.007
Group C	56	26.48 ± 0.68	21.49 ± 0.58^#,&^	22.98 ± 0.76^#,&^	26.02 ± 0.78	7.114	0.003
*F*		0.261	4.121	4.135	0.157		
*P*		0.412	0.021	0.022	0.572		

* Compared with Group A, *P *< 0.05.

^#^ Compared with Group B, *P *< 0.05.

^&^ Compared with 1 day before the operation, *P *< 0.05.

**Table 5:** Comparison of Montreal Cognitive Assessment (MoCA) scores of children before and after surgery.

Group	*n*	1 day before the operation	1 day after the operation	3 days after the operation	7 days after the operation	*F*	*P*
Group A	46	27.54 ± 0.53	21.79 ± 0.72^&^	24.63 ± 0.76^&^	26.11 ± 1.02	7.117	0.005
Group B	50	27.17 ± 0.75	23.36 ± 1.03^*,&^	26.01 ± 0.69^*,&^	26.62 ± 1.27	5.136	0.010
Group C	56	27.14 ± 0.82	21.12 ± 1.10^#,&^	24.67 ± 1.05^#,&^	26.37 ± 1.02	6.429	0.008
*F*		0.712	4.143	3.253	0.521		
*P*		0.289	0.026	0.036	0.321		

* Compared with Group A, *P *< 0.05.

^#^ Compared with Group B, *P *< 0.05.

^&^ Compared with 1 day before the operation, *P *< 0.05.

## 4 Discussion

In clinical anesthesia, tracheal catheter extraction during recovery is one of the the most risky procedures. Improper selection of tracheal catheter extraction time can compromise the recovery quality of children, lead to symptoms such as restlessness or respiratory depression, and even threaten the life safety of children (11, 12). Perioperative respiratory adverse events (PRAEs) are the most common complications observed in children receiving anesthesia. Children with PRAEs generally have minor (decreased oxygen saturation, airway obstruction, cough, or wheezing) and severe (laryngospasm and bronchospasm) adverse events (13–16). A considerable proportion of children undergoing tonsillotomy experience PRAEs, with a prevalence rate reaching 50% (17). The children enrolled in this study were divided into different groups based on the NT values, to explore the effect of tracheal extubation at different depths of anesthesia on the recovery quality from anesthesia and cognitive function of children undergoing tonsillotomy. Differences were observed in the awakening time and the pain score after awakening among the three groups, and the awakening time of the conscious group was significantly shorter than that of the light sedation and conventional sedation groups. An association was found between the awakening time and the depth of anesthesia. Children with light anesthesia experience a shorter time of consciousness recovery and awakening time undoubtedly, supporting that children in the conventional sedation group had the longest awakening time. In terms of pain score, the pain score after awakening in the conscious group was significantly higher than that in the light sedation and conventional sedation groups. However, no obvious difference was found between the light sedation and conventional sedation groups, indicating a good analgesic effect in both groups. Furthermore, the incidence of adverse reactions after extubation was 19 (41.30%) in the conscious group, with the highest rate of restlessness. The incidence of adverse reactions after extubation was 5 (10.00%) in the light sedation group, whereas the conventional sedation group had an incidence of adverse reactions after extubation of 15 (32.61%), with the highest proportion of hypoxemia. It might be related to the decrease of ventilation caused by the incomplete recovery of spontaneous respiration and swallowing reaction, glossocoma, and the retention of respiratory and digestive tract secretions (18). Generally, restlessness is a common postoperative complication in children, which may be related to the pain caused by intraoperative trauma, causing discomfort to the children (19). A lower performance of analgesic effect is expected for children with light anesthesia, leading to an increased proportion of restlessness. Shallow anesthesia may induce laryngeal spasms, but the incidence of laryngeal spasms was the same among the three groups observed in this study, which may be related to the fact that there were 46–56 patients in the three groups of samples selected in this study and that intraoperative cold incision had less damage to blood vessels and nerves, light wound bleeding and edema, and lower risk of blood aspiration to the respiratory tract. With the increase in the depth of anesthesia, children might also experience hypoxemia that might induce trauma to children, despite an increase in analgesic effect, which was not conducive to their postoperative rehabilitation (20). Under a certain anesthesia depth index interval, extubation can effectively avoid sudden awakening caused by shallow anesthesia depth (21, 22). Therefore, it is of great significance to balance the depth of anesthesia and determine a better NT value. In our study, the total incidence of adverse reactions in the light sedation group was lower than that in the conscious and conventional sedation groups. This suggests that light sedation has a better analgesic effect and a low incidence of adverse reactions. In addition, concerning cognitive function, no significant difference was found in the MMSE and MoCA scores before the operation and at 7 days after the operation among the three groups; a significant difference was noticed in these two scores at 1 day and 3 days after the operation among the three groups. These scores of the three groups reduced obviously at 1 day and 3 days after the operation than those of the three groups at 1 day before the operation. Furthermore, 3 days after the operation, the scores in the light sedation group were both higher than those in the other two groups. These data suggest that anesthesia during operation may have an adverse effect on the cognitive function of children to some extent. With postoperative recovery, there may be a gradual decrease in the adverse impact of anesthesia, with the fastest recovery after the operation in the light sedation group. At present, the mechanism of cognitive dysfunction in children remains unclear, which may be related to the method of anesthesia, the choice of intoxicants, and the timing of exposure (23, 24).

Although this clinical study took into account the short operation time and a small amount of blood loss, it is still necessary to further improve the index data of MAP, heart rate, and anesthesia time. Further large-scale, prospective, multicenter randomized controlled studies are needed to provide a more reliable basis for exploring the effects of tracheal catheter removal under different degrees of anesthesia on the recovery quality and cognitive function of children.

To sum up, our study suggests that compared with other NT values, tracheal extubation exhibits the smallest impact on the recovery quality from anesthesia and cognitive function of children undergoing tonsillotomy at the NT value of 80–94, which may contribute to the improvement in postoperative cognitive dysfunction and reduction of PRAE occurrence.

## Data Availability

The original contributions presented in the study are included in the article/Supplementary Material, and further inquiries can be directed to the corresponding author.
